# Environmental and Life-Style Related Risk Factors for Sinonasal and Nasopharyngeal Malignancies among a Prospective Cohort in Jos, Nigeria

**DOI:** 10.1155/2018/8524861

**Published:** 2018-10-16

**Authors:** Adeyi A. Adoga, Daniel D. Kokong, Tonga L. Nimkur, Nuhu D. Ma'an

**Affiliations:** ^1^Department of Otorhinolaryngology, Head and Neck Surgery, Faculty of Medical Sciences, University of Jos, PMB 2084, Jos, Plateau State, Nigeria; ^2^Department of Otorhinolaryngology, Head and Neck Surgery, Jos University Teaching Hospital, PMB 2076, Jos, Plateau State, Nigeria

## Abstract

**Background:**

Worldwide evidence indicates that environmental and life-style related factors are associated with increased risk for cancers in the head and neck region. We aim to study the association between these risk factors and cancers in the sinonasal and nasopharyngeal regions in our environment.

**Methods:**

Longitudinal prospective cohort study at the Jos University Teaching Hospital, Jos, Nigeria. Risk exposures were classified based on the International Agency for the Research on Cancer (IARC) classification of suspected carcinogens. Associations between variables were analyzed using logistic regression.

**Results:**

We studied 44 patients with malignancies in nasopharynx (n= 24; 54.5%) and sinonasal regions (n= 20; 45.5%). Male to female ratio is 1.9:1 and mean age is 45.2 years. Alcohol was the commonest risk factor in males (n= 19; 43.2%) while cooking wood fumes were the commonest in females (n= 14; 31.8%) which was associated with increased risk for malignancies for all sites, showing ten times risk in nasal cancers (OR= 9.67; 95% CI 1.87- 9.88; p= 0.01). Tobacco was associated with elevated risk of malignancies in the nasomaxillary and nasal regions. Other risks were herbicides, pesticides, and chemical fertilizers in farmers.

**Conclusion:**

The significant risk exposures in females were cooking wood fumes and alcohol, tobacco, and exposure to agricultural chemicals in males. Life-style modification and environmental changes to ensure clean air in Nigeria are essential to reduce risks.

## 1. Introduction

Cancers involving the sinonasal region are fairly uncommon representing approximately 0.2% to 1% of all cancers and 3% to 5% of all upper airway malignancies [1], exhibiting diverse histological subtypes, biology, and therapeutic response. They occur twice as many times in males than females usually above 40 years of age and present with nonspecific symptoms which mimic inflammatory diseases making early recognition difficult [2]. Nasopharyngeal cancers however constitute 0.7% of all cancers worldwide with one-third of the cases occurring in southern China and widely associated with Epstein-Barr virus (EBV) [3]. The risk factors for these cancers like most head and neck cancers are exposure to life-style related factors such as cigarettes and alcohol while exposure to environmental factors like hardwood and leather dust and dietary components like salted fish consumption are responsible for sinonasal and nasopharyngeal cancers, respectively [3, 4]. Other reported risk factors for these malignancies have been exposure to formaldehyde, textile dust in women and chromium in sinonasal malignancies [5] and exposure to incense smoke, wood combustion, use of herbal nasal medicine, and consumption of smoked meat associated with nasopharyngeal malignancies [6, 7]. However, the joint effects of this environmental and other nonenvironmental factors are reported to be responsible in the etiology of these malignancies [7].

The International Agency for the Research on Cancer (IARC) which is the World Health Organisation (WHO) specialized agency for cancer reported in 2013 that outdoor air pollution is carcinogenic to humans, classifying air pollution and particulate matter as IARC group 1 carcinogens signifying the worldwide impact of air pollution in cancer deaths [8]. In Nigeria like other developing countries of the world, environmental pollution due to rapid industrialization has become an increasingly prominent issue for concern as there are no emission controls making individuals in these parts of the world more susceptible to the carcinogenic effects of pollutants.

Studies have demonstrated that lifetime exposure to indoor solid fuel (coal and wood) burning used for cooking and heating was associated with an increased risk of head and neck cancers especially hypopharyngeal cancers and lung cancers [9]. Exposure to domestic woodfire in households without proper ventilation is associated with an increased risk of developing nasopharyngeal cancers among the Chinese [10] while an independent study conducted in southern Brazil demonstrated an increased risk of developing cancers particularly in the oral cavity, pharynx, and larynx with the use of wood stoves [11]. Live animal studies have demonstrated that particulate matter from air pollution has in vivo mutagenic effects on germline DNA in offspring who inherit these DNA mutations from their parents [12]. Studies in humans reveal that particulate matter from air pollution induce immune responses such as long-term inflammatory and oxidative stress in the upper aerodigestive tract particularly DNA or protein damage including DNA or protein adducts linked to cancer [13, 14].

The studies highlighted above support the evidence of an association of air pollution and cancers in the upper aerodigestive tract; however, studies from Nigeria on the association between these environmental risk factors and cancers involving the sinonasal and nasopharyngeal regions are lacking. To the best of our knowledge this is the first of such study from Nigeria. We hope that the findings will provide information to the possible risk factors for environmental modification and the reduction of risk exposure.

## 2. Patients and Methods

### 2.1. Study Design and Setting

This is a longitudinal prospective study of a cohort of patients with histologically confirmed sinonasal and nasopharyngeal malignancies managed at the Jos University Teaching Hospital a 520-bedspace tertiary referral hospital from February 2013 to January 2018.

### 2.2. Study Protocol

Approval for this study was provided by the Institutional Health Resource Ethics Committee (IHREC) of the Jos University Teaching Hospital.

Consecutive patients presenting with features of sinonasal and nasopharyngeal malignancies within the study period were included and studied for age, gender, occupation, presenting symptoms, duration of symptoms, exposure to risk factors and duration of exposure, treatment, histological diagnosis, and outcome of treatment. Thorough history of illness and examination of the patients were done at presentation with hematological and radiological investigations were necessary. Patients presenting in respiratory distress as a result of airway compromise had emergency tracheostomies. Elective tracheostomies were done for those in imminent obstruction and for securing the airway for administration of anesthesia at surgery in those with large tumors in whom oral and nasal endotracheal intubation was impossible. Treatment offered was surgical excisions, examination under anesthesia and biopsy, radiotherapy, and chemotherapy.

Standardized codes in the International Classification of Diseases, tenth revision (ICD-10), were used to class malignancies based on sites.

Risk exposures were classified based on the IARC classification that evaluates known and suspected carcinogens [15]. The risk exposures we considered for sinonasal and nasopharyngeal malignancies were alcohol consumption and tobacco use (which includes cigarette smoking, tobacco chewing, and inhaling snuff). Others were industrial fumes, wood dust, fumes from cooking wood, nickel dust, leather dust, herbicides, pesticides, and chemical fertilizer from farming exposure, soldering, and welding.

Data was entered into a predesigned structured format over the study period.

Patients with benign sinonasal and nasopharyngeal tumors were excluded from the study. Patients with head and neck malignancies other than the nasopharynx and sinonasal regions were also excluded.

Patients lost to follow-up were not included in the final data compilation.

### 2.3. Data Analysis

The data generated was entered into EPI Info statistical software ® version 7.2.2.1 (EPI Info, Center for Disease Control, Atlanta, Georgia, 2017) and analyzed.

Descriptive analysis of means ± standard deviation for continuous variables and the number of observations (%) for nominal variables were used to summarize the data. A p value of ≤ 0.05 was considered statistically significant. The associations between variables were modelled using logistic regression analysis in which odds ratios (OR) with 95% confidence intervals (CI) were used to assess the strength of association between risk factors and sinonasal and nasopharyngeal malignancies.

## 3. Results

### 3.1. Biodemographic Characteristics

A total of 44 patients presented with malignancies in the nasopharynx (n= 24; 54.5%) and the sinonasal region (n= 20; 45.5%) consisting in total of 29 (66.0%) males and 15 (34.0%) females (male to female ratio of 1.9:1) aged 13 years to 82 years (mean= 45.2 years; standard deviation= ±18.4); sinonasal (mean= 42.1 years; standard deviation= ±13.3); nasopharyngeal (mean= 44.0 years; standard deviation= ±17.6) with each malignancy commoner in males.Farming (n= 14; 31.8%) was the commonest occupation among the study population (Table 1).

### 3.2. Site and Clinical Features

The commonest cancer site was the nasopharynx (n= 24; 54.5%) with majority occurring in males. Malignancies involving the maxillary antrum alone were the commonest in the sinonasal region (Table 2). It is important to note that the exact origin of these malignancies could not be established as our patients had tumors that had involved one or more other anatomic sites at presentation.

The commonest presenting symptoms were nasal obstruction (n= 34; 77.3%) in patients with nasopharyngeal cancers (p= 0.03) and epistaxis (n= 25; 56.8%) in those with maxillary antral malignancies (0.05). The duration of symptoms before presentation was 1 month to 36 months (mean= 11.8; standard deviation= ±6.0). Cervical lymph node enlargement (n= 20; 45.5%) in nasopharyngeal cancers (p< 0.001) and presence of nasal mass (n= 18; 40.9%) in nasal cancers (p= 0.03) were the most recorded finding on clinical examination.

### 3.3. Risk Exposure

Alcohol was the commonest risk factor in males (n= 19; 43.2%) while exposure to cooking wood smoke was the commonest in females (n= 14; 31.8%) with 16 (36.4%) patients exposed to 2 or more risks. Eleven (78.6%) of the farmers were exposed to herbicides and pesticides while 9 (64.3%) were exposed to urea-based chemical fertilizers. Other risk exposures are shown on Table 3 with the mean durations of exposure. The pooled duration of risk exposure was 2 years to 55 years (mean= 27.2 years; standard deviation= ±16.5). The duration of alcohol consumption was 10 years to 55 years (mean= 33.7 years; standard deviation= ± 12.4). The duration of exposure to smoke from cooking wood was 5 years to 52 years (mean= 27.1 years; standard deviation= ±16.3) (Table 3).

Statistical analysis shows alcohol to be associated with an elevated risk for malignancies involving the sinonasal regions but not the nasopharynx. Exposure to cooking wood smoke was also associated with an increased risk for malignancies for all the sites studied especially malignancy in the nose showing almost ten times increased risk with an OR of 9.67 (95% CI 1.87-9.88; p= 0.01). Tobacco was involved with an elevated risk for malignancies in the nasomaxillary and nasal regions (Table 4).

### 3.4. Clinical Stage

Majority of our patients presented with stages II (n= 29; 66.0%) and III (n= 13; 29.5%) diseases, respectively. No patient presented with stage I disease.

### 3.5. Histologic Types

Most malignancies (86.4%) were of epithelial origin with squamous cell carcinomas accounting for 65.9% of the histologic types seen (Table 5).

### 3.6. Treatment and Outcome

Treatment was multimodal for all patients consisting of examination under anesthesia and biopsy for histological diagnosis, tracheostomy, surgical excisions with cosmetic repair, and postoperative radiotherapy and/or chemotherapy. All patients had surgery consisting of 12 (27.3%) tracheostomies of which 9 (20.5%) were emergencies. Twenty-two (50.0%) patients had examination under anesthesia and biopsy. Postoperative treatment in the form of radiotherapy (n= 40; 91.0%) and chemotherapy (n= 16; 36.4%) was done.

Disease recurrence has occurred in 3 (7.3%) patients, two patients who presented with stage IV and one with stage III diseases, respectively, the lungs being the commonest site in 2 (66.7%) of the cases. All patients are still being followed up with a total of 4 (9.1%) deaths so far recorded as a result of late stage presentation.

## 4. Discussion

This study evaluated the association between environmental and life-style related risk factors and malignancies occurring in the nose, nasopharynx, and the sinonasal regions.

The gender ratio in our study shows a preponderance of males for both malignancies in the sinonasal regions and the nasopharynx, similar to other studies in literature [2, 6, 10]. The male preponderance noted in our environment is because more males are exposed to risk factors such as smoking and alcohol than females. This is shown by the relative nonassociation of smoking and alcohol consumption and the female gender in our study

The most significant risk exposure among our study population of predominantly males for both sinonasal and nasopharyngeal malignancies presenting in their fifth decades of life was alcohol in 43.2% of patients with a mean exposure duration of 33.7 years. This was associated with a minimal increased risk for sinonasal and not nasopharyngeal malignancies. The use of alcohol and tobacco and exposure to ionizing radiation are not usually associated with an increased risk for sinonasal cancers as opposed to environmental and occupational exposures [4]. Our finding however shows that alcohol is associated with a minimal increased risk. However, smoking cigarettes has been reported to increase the risk of nasal cancers and the risk is doubled in heavy or long-term smokers with a risk reduction after long-term cessation of smoking. A significant dose-response relationship was also noted between alcohol drinking and risk of nasal cancer [16]. An increased risk for nasal cancers is noted in our study with patients exposed to tobacco and alcohol conforming to this report. Both alcohol and tobacco act independently and synergistically to cause genetic damage to squamous cells in the mucosa of the aerodigestive tract by the covalent bonding of the carcinogen benzopyrene diol epoxide (BPDE) and deoxyribonucleic acid (DNA) adducts throughout the genome including cytochrome p53 mutations [17].

Another significant risk in our study is the exposure to fumes from cooking wood which was associated with malignancies in the nasopharynx and all the sinonasal regions. Studies have demonstrated the elevated risks of malignancies in these anatomical regions with exposure to biofuels of this nature especially in improperly ventilated environments [9, 10]. This has been attributed to long-term inflammatory and oxidative stress in the upper aerodigestive tract particularly DNA or protein damage including DNA or protein adducts which are linked to cancers [13, 14]. Wood is the most affordable means of fuel for cooking in our environment and cooking is a practice more prevalent among women in the Nigerian culture which is buttressed by the higher female population with sinonasal malignancies in our study.

Wood dust exposure was associated with elevated risk for malignancies involving the sinonasal regions in our study. This exposure is implicated in adenocarcinomas in the sinonasal region in which the offending substances in the wood dust have been reported to be formic acid and hydrocarbon produced by pyrolysis of wood [2].

The largest group of patients we studied were farmers with significant exposure to herbicides, pesticides, and chemical fertilizers which contain nitrates used to improve farming yield. These are shown to be associated with an elevated risk for malignancies of the sinonasal region in our study. These agents are known and classed as carcinogens by the IARC [8]. In vitro studies using human nasal mucosal tissue have demonstrated the genotoxic effects of active substances in pesticides providing evidence of the potential carcinogenic effects of these agents to the human nasal mucosal cells [18]. The system of enumerating costs and benefits in the use of herbicides and fertilizers which contain toxic agents should be discouraged and research into the production of safer alternatives should be encouraged in other to reduce risks.

Hydrocarbons emitted by exhaust fumes from diesel engines and other fossil fuels and fine particulate materials which are prevalent in our environment constitute significant risk exposures which are classed as carcinogens by the International Agency for Research on Cancer (IARC). These are risks that individuals in our society are exposed to daily for which government regulations on air pollution and exposure are nonexistent. We did not establish the relationship between these carcinogens and malignancies in the sinonasal and nasopharyngeal regions in the present study. These relationships require further evaluation in order to reduce risks.

Several patients in our study were exposed to two or more known risk factors and it is known that the joint effects of this environmental and other nonenvironmental factors are reported to be responsible in the etiology of these malignancies [7]. Genetic factors also play significant roles in the etiology of cancers. Life animal studies have shown an inheritable mutagenic effect of environmental pollution in which it was concluded that exposure to environmental pollution induces DNA mutations which offspring inherit from their parents [12] therefore predisposing to the development of cancers. Additional research on the role of multiple risk factors in our environment is required in understanding how to prevent these cancers.

As further research is required in the understanding of the biology of life-style related and environmental pollution-associated cancers, it is important to modify the environment to reduce pollution by the use of clean energy for example in cooking. European countries have continued to make concerted efforts to achieve this by adhering to the WHO guidelines to clean air [19]. These efforts have not been made by Nigeria as the political will is lacking. We therefore suggest a commitment by Nigerians in adhering to these guidelines spearheaded by the government via legislation, health education, and provision of support programs to achieve environmental protection and occupational safety for citizens at risk.

## 5. Conclusion

The identified environmental-associated risk factor for malignancies in the nose, nasopharyngeal, and sinonasal regions in females especially in the fifth decade was exposure to cooking wood fumes while alcohol, tobacco, and exposure to agricultural chemicals were significant risk exposures in males.

Health education on life-style modification and environmental changes to achieve clean air in Nigeria are essential to reduce the risks of these malignancies.

## Figures and Tables

**Table 1:** Sociodemographic characteristics of study population (n=44) by tumor sites.

**Age group (yrs)**	**NPC**	**Sinonasal**				**Total**
		**MA**	**NC**	**NM**	**NE**	
0-9	0	0	0	0	0	0
10-19	1	0	1	1	0	3
20-29	6	2	0	0	1	9
30-39	3	0	1	0	0	4
40-49	3	1	1	1	0	6
50-59	4	3	0	2	0	9
60-69	5	2	0	1	0	8
70-79	2	0	0	1	1	4
80-89	0	0	0	1	0	1
**Total**	**24**	**8**	**3**	**7**	**2**	**44**

**Occupation**						
Farmer	9	3	0	2	0	14
Housemaker	5	2	0	1	0	8
Retiree	0	0	0	1	0	1
Student	4	1	1	1	0	7
Trader	2	1	2	2	1	8
Carpenter	0	1	0	0	0	1
Civil servant	2	0	0	0	0	2
Nurse	1	0	0	0	0	1
Unemployed	1	0	0	0	1	2
**Total**	**24**	**8**	**3**	**7**	**2**	**44**

NPC: nasopharyngeal cancer; MA: maxillary antrum; NC: nasal cavity; NM: nasomaxillary; NE: nasoethmoidal.

**Table 2:** Tumor site distribution by gender.

**Tumor site (Diagnostic code)**	**Gender**		**Total (**%**)**
	**Male (**%**)**	**Female (**%**)**	
Maxillary antrum (C31.0)	6 (20.7)	2 (13.3)	8 (18.2)
Nasal cavity (C30.0)	3 (10.3)	0 (0)	3 (6.8)
Nasomaxillary (C31.0)	3 (10.3)	4 (26.7)	7 (16.0)
Nasoethmoidal (C80.0)	1 (3.5)	1 (6.7)	2 (4.5)
Nasopharynx (C11.0)	16 (55.2)	8 (53.3)	24 (54.5)
**Total**	**29 (100)**	**15 (100)**	**44 (100)**

**Table 3:** Risk exposures by gender.

**Risk**	**Male**	**Female**	**Exposure duration in years (Mean/Standard deviation)**
Alcohol	19	0	33.7/ 12.4
Cooking wood	2	14	27.1/ 16.3
Fertilizer	9	1	30.3/ 14.5
Herbicides	11	5	27.2/ 16.5
Pesticides	6	4	20.4/ 12.6
Tobacco	4	0	25.3/ 13.9
Wood dust	1	0	15.0/ 16.6

**Table 4:** Multivariate logistic regression showing odds ratios (95% CI) and p-values of covariates as risks for malignancy.

Variable (Risk)	Sites				
	NP	MA	NC	NM	NE
Alcohol	1.86(0.55-6.28;p=0.83)	0.75(1.16-3.60;p=0.01)	0.64(1.05-7.62;p=<0.01)	0.60(1.13-2.76;p=0.01)	0.63(0.33-1.20;p=0.1)
Cooking wood	0.05(1.22-2.57;p=0.02)	1.01(1.61-12.17;p=<0.01)	9.67(1.87-9.88;p=0.01)	2.73(1.61-12.17;p=<0.01)	1.67(1.95-2.95;p=0.04)
Fertilizer	1.20(0.27-5.26;p=0.81)	8.0(1.41—45.23;p=0.02)	8.67(2.25-35.0;p=0.04)	6.20(1.16-33.17;p=0.02)	0.24(1.12-4.11;p=0.04)
Herbicides	1.13(0.22-5.78;p=0.63)	2.07(1.32-13.27;p=<0.01)	11.67(2.91-49.7;p=0.05)	0.10(1.01-12.6;p=0.03)	4.71(1.56-39.4;p=<0.01)
Pesticides	0.24(0.13-4.42p=0.07)	0.80(1.00-1.35;p=0.24)	0.60(1.03-3.72;p=0.03)	0.33(0.62-1.92;p=0.16)	0.36(0.37-2.10;p=0.82)
Tobacco	0.25(0.02-2.58;p=0.24)	0.62(0.19-2.0;p=0.4)	6.32(1.44-9.80;p=<0.01)	1.40(0.80-2.47;p=0.2)	0.28(0.18-1.41;p=0.06)
Wood dust	0.01(0.20-11.45;p=0.42)	4.80(2.95-24.23;p=0.05)	0.01(1.30-9.14;p=<0.01)	4.66(1.10-13.05;p=0.04)	0.71(0.40-1.01;p=0.4)

CI: confidence interval; NP: nasopharynx; MA: maxillary antrum; NC: nasal cavity; NM: nasomaxillary; NE: nasoethmoidal.

**Table 5:** Histopathologic type.

**TYPE**	**NPC**	**Sinonasal**				
		**MA**	**NC**	**NM**	**NE**	**Total (**%**)**
Squamous cell carcinoma	20	3	3	2	1	29 (65.9)
Adenocarcinoma	-	1	-	-	1	2 (4.5)
Adenoid cystic carcinoma	-	2	-	1	-	3 (6.8)
Mucoepidermoid carcinoma	-	-	-	1	-	1 (2.3)
Undifferentiated carcinoma	1	1	-	-	-	2 (4.5)
Osteogenic sarcoma	-	1	-	-	-	1 (2.3)
Craniopharyngioma	1	-	-	-	-	1 (2.3)
Lymphoma	1	-	-	1	-	2 (4.5)
Rhabdomyosarcoma	1	-	-	-	-	1 (2.3)
Plasmacytoma	-	-	-	1	-	1 (2.3)
Small blue cell tumor	-	-	-	1	-	1 (2.3)

NPC: nasopharyngeal cancer; MA: maxillary antrum; NC: nasal cavity; NM: nasomaxillary; NE: nasoethmoidal.

## Data Availability

The data used to support the findings of this study are available from the corresponding author upon request.
